# The Molecular Mechanism of Chronic High-Dose Corticosterone-Induced Aggravation of Cognitive Impairment in APP/PS1 Transgenic Mice

**DOI:** 10.3389/fnmol.2020.613421

**Published:** 2021-01-15

**Authors:** Shen-Qing Zhang, Long-Long Cao, Yun-Yue Liang, Pu Wang

**Affiliations:** College of Life and Health Sciences, Northeastern University, Shenyang, China

**Keywords:** Alzheimer’s disease, corticosterone, glucocorticoid receptor, BACE-1, CREB, apoptosis, neuronal differentiation

## Abstract

Clinical studies have found that some Alzheimer’s disease (AD) patients suffer from Cushing’s syndrome (CS). CS is caused by the long-term release of excess glucocorticoids (GCs) from the adrenal gland, which in turn, impair brain function and induce dementia. Thus, we investigated the mechanism of the effect of corticosterone (CORT) on the development and progression of AD in a preclinical model. Specifically, the plasma CORT levels of 9-month-old APP/PS1 Tg mice were abnormally increased, suggesting an association between GCs and AD. Long-term administration of CORT accelerated cognitive dysfunction by increasing the production and deposition of β-amyloid (Aβ). The mechanism of action of CORT treatment involved stimulation of the expression of BACE-1 and presenilin (PS) 1 in *in vitro* and *in vivo*. This observation was confirmed in mice with adrenalectomy (ADX), which had lower levels of GCs. Moreover, the glucocorticoid receptor (GR) mediated the effects of CORT on the stimulation of the expression of BACE-1 and PS1 *via* the PKA and CREB pathways in neuroblastoma N2a cells. In addition to these mechanisms, CORT can induce a cognitive decline in APP/PS1 Tg mice by inducing apoptosis and decreasing the differentiation of neurons.

## Introduction

Clinical studies have found that Alzheimer’s disease (AD) patients usually suffer from corticosteronism, which is characterized by the secretion of high levels of glucocorticoids (GCs) from the adrenal cortex (Guldiken and Guldiken, 2008; Haraguchi et al., 2016). In an A mouse model of an anxiety/depression-like state, long-term exposure to GCs results in structural changes in the brain, similar to those observed in aging (David et al., 2009; Herbert and Lucassen, 2016). In detail, the long-term administration of GCs can impair specific cognitive regions, such as the neocortex and hippocampus (Starkman et al., 2001). When the cortisol dropped to the basal level, the size of the hippocampus will be enlarged accompanied by improving the learning ability (Starkman et al., 2001, 2003). Reciprocally, chronic high levels of GC lead to a decrease in the volume of the hippocampus, which results in impairing cognitive ability (Starkman et al., 1992; Sheline et al., 1996; Bettio et al., 2017). More closely, patients with high levels of GC show long-term impairment of memory and concentration (Ragnarsson et al., 2012). Based on these prior works, GCs potentially contribute to accelerating the progression of AD.

The production of GCs is tightly regulated under physiological conditions to avoid pathological effects. GCs is a self-regulating molecule in the endocrine system and is responsible for modulating stress reaction. GCs are synthesized and secreted by the fascicular zone of the adrenal cortex. GCs can be classified into two categories, hydrocortisone (HC) and cortisone, the synthesis and secretion of which are regulated by the hypothalamic-pituitary-adrenal (HPA) axis (Turnbull and Rivier, 1999). In detail, the nucleus of the paraventricular hypothalamus releases corticotropin-releasing factor (CRF), which promotes the secretion of adrenocorticotropic hormone (ACTH) by acting on the anterior pituitary, leading to the synthesis of GCs by the fascicular zone of the posterior adrenal cortex (Curran and Chalasani, 2012). GCs is primarily synthesized and secreted from the adrenal cortex and penetrates the blood-brain barrier (BBB) to function in the central nervous system (CNS).

The physiological and pharmacological functions of GCs are mediated by the intracellular glucocorticoid receptor (GR), which belongs to the family of nuclear receptors. Thus, the GR can promote or inhibit the transcription of target genes by directly binding to the glucocorticoid response element (GRE) or interacting with other transcription factors (Ratman et al., 2013; Meijer et al., 2018). Mineralocorticoid receptors (MR) have higher GCs binding activity than the GR (Richardson et al., 2016; Faught and Vijayan, 2019). Under resting conditions, the level of GCs is relatively low and GCs mainly bind to MR. When the level of GCs is elevated under pathological conditions, GCs bind to and activate the GR to induce biological effects (Brinks et al., 2007). In AD patients, GR-expressing neurons undergo progressive atrophy and loss, resulting in a decrease in the expression of the GR, leading to the excessive loading of GCs by disrupting the negative feedback of the GR on the HPA axis that regulates GCs synthesis (Sapolsky et al., 1986; Jacobson and Sapolsky, 1991). Also, overexpression of the GR in male C57BL/6L mice accelerates the aging phenotype, including neuroendocrine dysregulation and deficit in cognitive function (Wei et al., 2007). Specifically, blocking the GR for only 3 days in 12-month-old APP/PS1 Tg mice reduced the production of Aβ_1–40_ and Aβ_1–42_ in the hippocampus, resulting in the rescue of cognitive deficit (Lesuis et al., 2018). These observations were confirmed by reports that showed that treatment with a GR antagonist (mifepristone) completely reversed synaptic deficits and hippocampal apoptosis and partially reversed cognitive deficit, which are effects of the hippocampal amyloidogenic pathway and neuroinflammation (Pineau et al., 2016). Also, selective GR modulators can reverse hippocampal Aβ generation, neuroinflammation, and apoptosis; restore the hippocampal levels of synaptic markers; and improve cognitive function (Pineau et al., 2016).

GCs have certain GR-independent effects on the regulation of AD development. For instance, administration of stress-level GCs increases the formation of Aβ by increasing the steady-state levels of amyloid precursor protein (APP) and β-APP-cleaving enzyme (BACE-1) in APP/PS1/tau^P301L^ Tg mice (Green et al., 2006). This process is mediated by lipid raft-dependent CREB activation (Choi et al., 2017). Additionally, GCs considerably reduce the degradation and clearance of Aβ by astrocytes, inducing a decrease in the neuroprotective ability of astrocytes (Wang et al., 2011). GCs act *via* these mechanisms to impair learning and memory by inducing apoptosis of neurons (Li et al., 2010).

Based on these considerations, we aimed to examine the multiple roles of GCs in the production and deposition of Aβ, neuronal apoptosis, and neuronal differentiation. The results demonstrate that GCs significantly increase the production of Aβ by enhancing the expression of BACE-1 and PS1. Moreover, GCs induce neuronal apoptosis by reducing the ratio of Bcl-2 and Bax and inhibiting neuronal differentiation. These effects eventually induce a cognitive decline in APP/PS1 Tg mice.

## Materials and Methods

### Reagents

Corticosterone (CORT) was obtained from Solarbio Life Sciences (Beijing, China). A PKA inhibitor, H89; an antibody specific for Aβ (A8354, mouse, dilution 1:100 for immunohistochemistry, IHC); and secondary antibodies were purchased from Sigma–Aldrich (St. Louis, MO, USA). Antibodies against APP (ab241592, rabbit, 1:2,000 for Western blot analysis), BACE1 (ab2077, rabbit, 1:2,000 for Western blot analysis; 1:100 for IHC and immunofluorescence, IF) and NeuN (ab104224, mouse, 1:100 for IF) were obtained from Abcam (Cambridge, MA, USA). Fluorescence-tagged secondary antibodies (A32727 mouse, A32732 rabbit, A11034 rabbit, A11029 mouse, 1:500 for IF) were purchased from Thermo Fisher Scientific (Waltham, MA, USA). Other antibodies, including ADAM10 (#14194, rabbit, 1:2,000 for Western blot analysis), PS1 (#5643, rabbit, 1:2,000 for Western blot analysis; 1:100 for IHC and IF), PS2 (#2192, rabbit, 1:2,000 for Western blot analysis), PEN2 (#5451, rabbit, 1:2,000 for Western blot analysis), nicastrin (#5665, rabbit, 1:2,000 for Western blot analysis), GR (#3660, rabbit, 1:2,000 for Western blot analysis; 1:100 for IF), CREB (#4820, rabbit, 1:2,000 for Western blot analysis), p-CREB (#9196, mouse, 1:2,000 for Western blot analysis), Bcl-2 (#3498, rabbit, 1:2,000 for Western blot analysis), Bax (#2774, rabbit, 1:2,000 for Western blot analysis), doublecortin (#14802, rabbit, 1:100 for IHC) and β-actin (#3700, mouse, 1:5,000 for Western blot analysis), were from Cell Signaling Technology (Danvers, MA, USA). All reagents for the sodium dodecyl sulfate-polyacrylamide gel electrophoresis (SDS–PAGE) experiments were purchased from Bio-Rad Laboratories (Hercules, CA, USA). All other reagents were from Thermo Fisher Scientific/Invitrogen (Waltham, MA, USA) unless specified otherwise.

### Cell Culture and Treatment

Neuroblastoma N2a cells were cultured in DMEM with 10% FBS in a 37°C cell incubator containing 5% CO_2_. Before treatment with corticosterone (CORT) or H89, the cells were cultured in a serum-free medium for 12 h. After changing the serum-free medium, 1 μM CORT or 10 μM H89 was added to the fresh medium. After 24 h, the cells were lysed to extract protein or RNA.

### Plasmid Construction

The primers for gRNA generation were synthesized by Genewiz, Inc. (Suzhou, Jiangsu, China). The sequences of the primers were as follows: NR3C1-1 forward, 5′-CACCGGCTTTGGATAAATCTGGCTG-3′; reverse, 5′-AAACCAGCCAGATTTATCCAAAGCC-3′; NR3C1-2 forward, 5′-CACCGGGATCATCTTCTCCCGCCAA-3′; reverse, 5′-AAACTTGGCGGGAGAAGATGATCCC-3′; and NR3C1-3 forward, 5′-CACCGCCAGCAGTTTGCTTGGCCGG-3′; reverse, 5′-AAACCCGGCCAAGCAAACTGCTGGC-3′. The paired forward and reverse primers were annealed in NE buffer 2.1 (New England Biolabs, Beverly, MA, USA). LentiCRISPR V2 plasmid (Addgene, Cambridge, MA, USA) was linearized by incubation at 37°C with BsmBI restriction enzymes for 2 h. The annealed primers were used as insertion fragments, which were then inserted into the linearized LentiCRISPR v2 plasmid by incubating with T4 ligase at 16°C for 2 h. The constructed plasmids were transferred to Stbl3 competent cells for amplification and subsequent Sanger sequencing.

### Transfection

LentiCRISPR v2 plasmids (6 μg) encoding gRNA of NR3C1 were cotransfected with 5 μg of psPAX2 and 3 μg of pMD2.G into HEK293T cells using Neofect (Neofect Biotech Company Limited, Beijing, China) transfecting reagent. The medium was replaced after 12 h. The virus was collected after 48 and 72 h of transfection by centrifugation at 8 × 10^4^ rpm. The N2a cells were then infected with the virus in the presence of 10 μg/ml polybrene (Merck Millipore, Billerica, MA, USA). After 48 h, the cells were selected in the presence of 3 μg/ml puromycin to establish NR3C1 knockdown cells.

### Animals and Treatment

**Figure 1:**
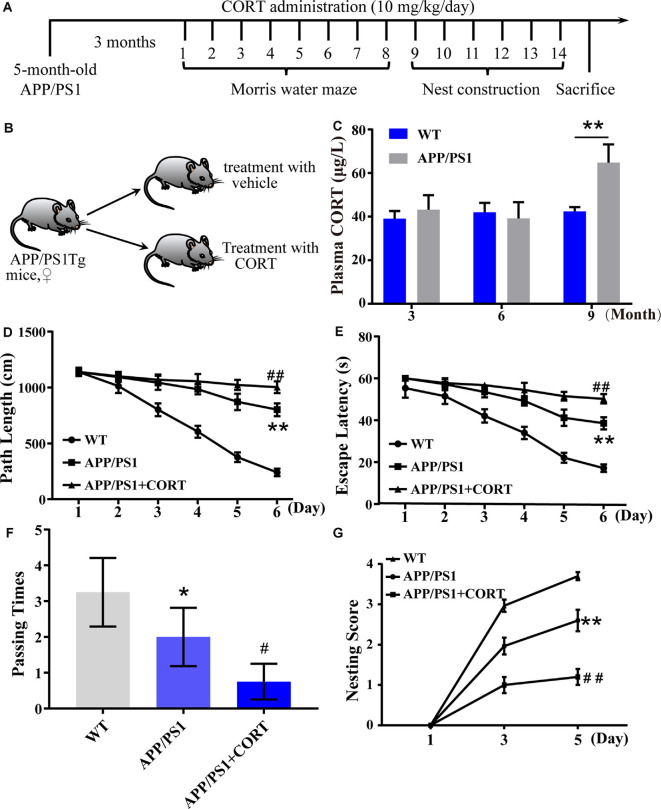
Elevated plasma levels of corticosterone (CORT) result in impaired learning in APP/PS1 Tg mice. **(A)** Timeline of the treatment of APP/PS1 Tg mice with CORT. **(B)** Schematic diagram of the random grouping and treatment of mice.** (C)** Plasma was collected from 3-, 6- and 9-month-old APP/PS1 Tg and age-matched WT mice. The content of CORT was determined by enzyme-linked immunosorbent assay (ELISA). The data were analyzed by repeated-measures ANOVA (*n* = 6). **(D,E)** APP/PS1 Tg mice (5-month-old) were intraperitoneally injected with CORT (10 mg/kg/day) for 3 months. **(D,E)** The escape distance and latency of mice were observed in the hidden platform experiment. **(F)** In the space exploration experiment, the crossing times to the original platform were recorded by the software. The data were analyzed by repeated-measures ANOVA (*n* = 6). **(G)** The nesting score of various groups of mice was analyzed by the nest construction assay. The data were analyzed by a nonparametric statistical test. The data are presented as the mean ± SE of independent experiments (*n* = 6). *p < 0.05; **p < 0.01 compared with the WT mice. ^#^*p* < 0.05; ^##^*p* < 0.01 compared with vehicle-treated APP/PS1 Tg mice.

APP/PS1 Tg mice were obtained from Jackson Laboratory (Bar Harbor, ME, USA). C57BL/6J mice were purchased from the Liaoning Changsheng Biotechnology Company Limited (Benxi, Liaoning, China). All mice were housed under the same conditions at standard room temperature and humidity in a light and dark cycle-controlled environment. To ensure that mice could freely move, eat, and drink, three to five mice were housed per cage. APP/PS1 Tg mice were used as a model for the AD study. The WT or APP/PS1 Tg mice at the age of 5 months were subcutaneously administered with CORT (10 mg/kg/day) for 3 months before assessment of their memory and physiological and biochemical parameters; the timeline and group sorting are shown in Figures 1A,B.

### Adrenalectomy

The WT or APP/PS1 Tg mice were fasted overnight before surgery and anesthetized by tribromoethanol (Sigma–Aldrich, Beijing, China). Mice were fixed in a prone position, and the long hair from the middle of the back was removed. A 1.5 cm midline incision was made, and muscle was exfoliated layer by layer from the outside to the inside until the organs were visible. The spherical adrenal tissue around the kidney was identified and gently removed with scissors, which was then placed back in the body before closing the wound. The other side of the adrenal gland was removed using the same method. The sham operation group only received an incision, and the skin was sutured. From the 3rd day after adrenalectomy (ADX) surgery, intraperitoneal injection of CORT (10 mg/kg/day) was performed for 7 days. Then, tribromoethanol was used for anesthesia, and the animals were sacrificed to collect the cerebral cortex and hippocampus.

### qRT-PCR

Total RNA was extracted by RNA isolator total RNA extraction reagent (Nanjing Vazyme Medical Technology Company Limited, Nanjing, Jiangsu, China). Purified RNA was reverse-transcribed into cDNA by a reverse transcription kit (Promega, Madison, WI, USA). qRT-PCR was performed according to the instructions of the qRT-PCR kit (Promega, Madison, WI, USA). The primer sequences for qRT-PCR were as follows: ADAM10 forward, 5′-CTCAAGCTTCGAAT TCATGGTGTTGCCGACAGTGTT-3′, reverse, 5′-GCGACCGGTGGATCCTTGCGTCGCATGTGTCCCAT-3′; BACE1 forward, 5′-CTCAAGCTTCGAATTCCAAGGCCCGGGCTCACTATG-3′, reverse, 5′-GGCGACCGGTGGATCCGCCTTGAGCAGGGAGATGTCATCA-3′ PS1 forward, 5′-CTCAAGCTTCGAATTCCTCCAATGACAGAGATACCTG-3′, reverse, 5′-GGCGACCGGTGGATCCGCGATATAAAACTGATGGAATG-3′; GAPDH forward, 5′-TGCAGTGGCAAAGTGGAGAT-3′, reverse, 5′-TTTGCCGTGAGTGGAGTCATA-3′. GAPDH was used as a housekeeping reference gene. The ratio was calculated as the following equation:

$$\text{Ratio}=\frac{2^{\Delta Ct({\text{Gene}}_{\text{wt}}-{\text{Gene}}_{\text{Tg}})}}{2^{\Delta Ct({\text{GAPDH}}_{\text{wt}}-{\text{GAPDH}}_{\text{Tg}})}}$$

The WT was always set to 1, and the value of the Tg mice was obtained from the previous equation.

### Western Blots

Cells or tissues of the cerebral cortex and hippocampus were homogenized and lysed on ice in RIPA buffer [25 mM Tris-HCl (Ph 7.6), 150 mM NaCl, 1% NP-40, 1% sodium deoxycholate, and 0.1% SDS], containing protease inhibitor cocktail (Thermo Scientific-Pierce, Rockford, IL, USA). The supernatant was collected after centrifugation at 13,000 *g*. The concentration of protein in the supernatant was determined by a BCA kit (Beyotime Biotechnology, Shanghai, China). Protein (2 μg/μl) was loaded onto SDS–PAGE gels and, following SDS–PAGE, was transferred to a PVDF membrane. The membranes were incubated with a primary antibody at 4°C overnight. The membranes were washed with TBST five times for 5 min each time. Then, the corresponding secondary antibody was added to the membranes and incubated at room temperature for 2 h. The membranes were probed with an antibody, and the bands were visualized by an ECL kit (Thermo Fisher Scientific, Waltham, MA, USA). β-Actin was used as an internal reference control.

### Immunohistochemistry (IHC)

The brains of WT or APP/PS1 Tg mice were fixed with 4% paraformaldehyde and dehydrated in a 30% sucrose solution. Sections (30 μm thickness) were prepared by a push slicer (Leica, Wetzlar, Germany). The slides were submerged in 3% hydrogen peroxide, to eliminate endogenous peroxidase activity. The sections were stained with antibodies specific for Aβ, BACE1, PS1, or doublecortin at 4°C overnight. A biotin-conjugated secondary antibody was then incubated with the sections for 30 min at room temperature after rinsing three times with PBS. The sections were then stained using a DAB solution [7 mg DAB + 50 ml Tris-HCl (pH 7.6) + 12 μl H_2_O_2_]. Before mounting, the slides were dehydrated with graded ethanol and were washed with xylene for 20 min. Finally, the sections were observed and imaged under an optical microscope (Leica, Wetzlar, Germany) using a 10× or 20× objective lens.

### Immunofluorescence

The cells or cerebral cortex and hippocampus tissue samples were fixed with 4% paraformaldehyde, permeabilized with 50 μg/ml digitonin for 10 min, and washed with PBS three times. The slides were blocked with 4% goat serum and incubated with a primary antibody specific to NeuN or GR at 4°C overnight. Then, the sections were incubated with Alexa 555-anti-rabbit IgG (1:500), Alexa 488-anti-rabbit IgG (1:500), and DAPI (1:1,000) for 1 h at room temperature. After rinsing, the sections were mounted using a fluorescent mounting reagent (Beyotime Biotechnology, Shanghai, China). The sections were visualized and imaged under a confocal microscope (Leica, Wetzlar, Germany) equipped with a 40× or 63× oil-immersion objective lens.

### Enzyme-Linked Immunosorbent Assay (ELISA)

The production of Aβ_1–42_ was determined by enzyme-linked immunosorbent assay (ELISA) kits (Thermo Fisher Scientific, Waltham, MA, USA). In brief, 50 μl of the standard curve storage solution and the sample solution were added to 96-well plates. Then, an Aβ_1–42_ antibody was added to the plate. After rinsing with wash buffer, the HRP-conjugated secondary antibody was incubated with the samples. After another rinse, the stabilized chromogen TMB was added to the plate for 30 min before measurement at 450 nm in a microplate reader.

CORT was assayed by ELISA kits (Wuhan Enzyme Immuno-Biotechnology Company Limited, Wuhan, Hubei, China) according to the manufacturer’s protocol. Briefly, 50 μl of the standard curve storage solution and the sample solution were added to 96-well plates. Enzyme-labeled reagent (50 μl) was added to the plates, which were incubated at 37°C for 30 min. After washing, 50 μl of developer A and developer B were added to the wells. After a 10 min incubation at 37°C, 50 μl stop solution was added to the wells before determination of the optical density of the CORT signal at 450 nm in a microplate reader.

### Morris-Maze Test

APP/PS1 Tg mice at the age of 5 months were treated with CORT (10 mg/kg/day) for 3 months. The Morris maze test was used to determine spatial learning and memory. In brief, mice were placed into a 1.6 m diameter pool, which had labeled quadrants, I, II, III, and IV. Milk was added to the pool, and the water was then heated to 25 ± 2°C. In the first 2 days, the platform was set 1–2 cm above the water level in quadrant II. The time and distance required for mice to find the platform was recorded for 60 s. If a mouse was unable to find the platform in 60 s, the mouse was guided to the platform or placed on the platform and kept on the platform for an additional 10 s. Starting from day 3, the platform was submerged under the water. The time and distance required for mice to find the platform was recorded in the following 4 days. On day 7, the platform was removed, and the number of crosses to the original location of the platform was recorded for 60 s. The experimental timeline is shown in Figure 1A.

### Nest Construction

After the Morris water maze test, each mouse was housed in a separate cage. Ten pieces of five 5 cm^2^ filter paper were placed in the cage. Each mouse was imaged for 6 days at the same time every day. The specific evaluation rules were as follows: 0, no nesting behavior of mice and no tearing or moving of paper; 1, no obvious nesting behavior in mice, and only slight tearing and moving of paper; 2, no nesting behavior in mice, and obvious tearing and moving of paper; 3, nesting behavior in mice, and most of the paper was torn into wicker shapes; and 4, obvious nesting behavior in mice, and all the paper was torn and moved to a corner. Finally, the mice were anesthetized and sacrificed using tribromoethanol to collect the cerebral cortex and hippocampus.

### Statistical Analysis

All data are presented as the mean ± SE, of at least three independent experiments. Data from the Morris water maze test and detection of CORT in plasma were analyzed using repeated-measures ANOVA. The paired *t*-test was used to analyze the nest construction data. Statistical significance of the differences between means was determined using Student’s *t*-test or one-way analysis of variance when appropriate.

## Results

### The Levels of CORT Are Elevated in the Plasma of APP/PS1 Tg Mice

In patients with sporadic AD, the plasma level of cortisol is increased in the early and late stages of the disease (Umegaki et al., 2000). The secretion of GCs is regulated by the HPA axis (Turnbull and Rivier, 1999). However, it is not known whether HPA axis dysfunction is the initial cause of AD. To detect whether the basic level of GCs is changed in AD animal models and whether the metabolic pathway of amyloid can change the production of GCs, the plasma CORT level of APP/PS1 Tg mice of various ages was detected. According to the data provided by Jackson Laboratory, APP/PS1 Tg mice express chimeric Mo/HuAPP695swe and mutant PS1-dE9 genes, Aβ is deposited in the brain of 6-month-old mice. Based on this information, plasma samples of wild-type (WT) and Tg mice were collected to determine the levels of CORT at the ages of 3, 6, and 9 months. The results demonstrated that the plasma levels in 3- and 6-month-old APP/PS1 animals were not significantly different from those of WT mice. In contrast, the plasma levels of 9-month-old APP/PS1 Tg mice were higher than those of the corresponding WT controls (Figure 1C). This observation suggests that enhanced production of GCs is associated with amyloidosis.

### CORT Exposure Accelerates the Cognitive Decline of APP/PS1 Tg Mice

To determine whether CORT can promote the pathological process in AD model mice, 5-month-old APP/PS1 Tg mice were intraperitoneally injected with CORT at a dose of 10 mg/kg/day for 3 months. The Morris maze test was performed to determine the learning and memory ability of mice. The results showed that mice treated with GCs take more time and travel a longer distance to find the hidden platform compared to untreated APP/PS1 Tg mice (Figures 1D,E). In the space exploration experiment, the crossing times of GCs-treated mice were considerably lower than those of vehicle-treated controls (Figure 1F), suggesting that GCs impairs the leaning ability of APP/PS1 Tg mice.

The nest-building test is an experimental method to assess the social ability of mice. The results can reflect the instinctive learning and social ability of mice based on biting paper and constructing a nest. In the nesting experiment, the nesting ability of mice treated with CORT for 3 months was significantly decreased compared with that of vehicle-treated controls (Figure 1G). In conclusion, CORT can accelerate the decline in learning and memory ability in mice, suggesting that a high level of GCs can accelerate the occurrence and development of AD *in vivo*.

### CORT Treatment Enhances the Deposition of Aβ in the Brain of APP/PS1 Tg Mice

**Figure 2:**
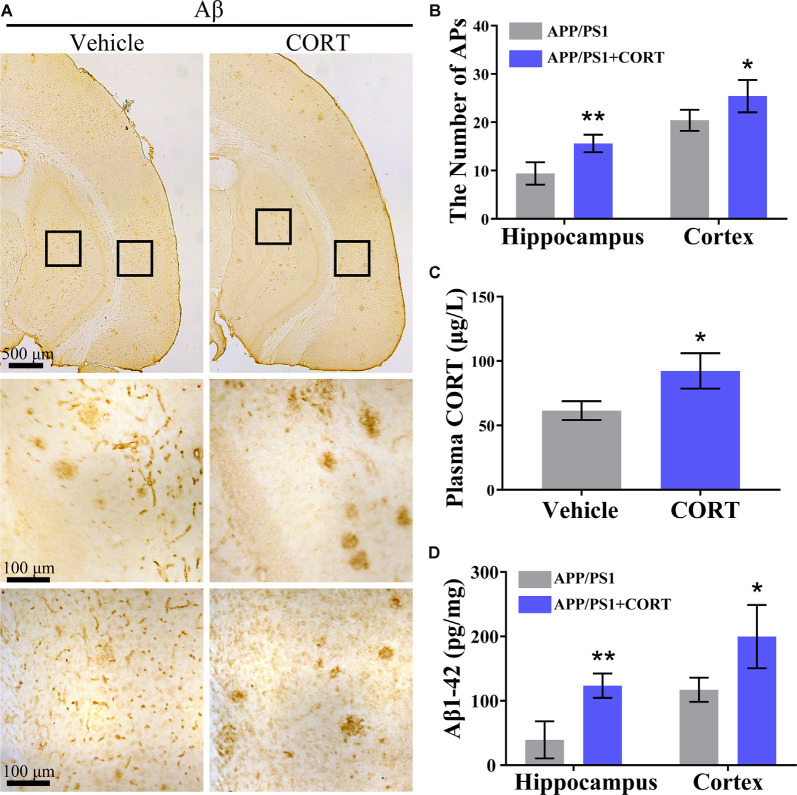
CORT treatment increases the production of Aβ and the number of APs in the brain of APP/PS1 Tg mice. APP/PS1 Tg mice (5-month-old) were intraperitoneally injected with CORT (10 mg/kg/day) for 3 months. **(A,B)** The distribution of APs in the cerebral cortex and hippocampus of APP/PS1 Tg mice was determined by immunohistochemistry (IHC). **(C)** The content of CORT was determined by ELISA. **(D)** The contents of Aβ_1–42_ in the cerebral cortex and hippocampus of APP/PS1 Tg mice were detected by ELISA. The data are present as the mean ± SE of independent experiments (*n* = 6). *p < 0.05; **p < 0.01 compared with the vehicle-treated mice by *t*-test.

According to Aβ theory, abnormal production and deposition of Aβ in the APs of the cerebral cortex and hippocampus is the key cause of the onset of AD (O’Brien and Wong, 2011). Thus, the effects of CORT on the production and deposition of Aβ in APP/PS1 Tg mice were assessed. APP/PS1 Tg mice (5-month-old) were intraperitoneally injected with CORT at a dose of 10 mg/kg/day for 3 months. The formation of APs was determined by IHC in CORT-treated APP/PS1 Tg mice. The results showed that the number of APs in the cerebral cortex and hippocampus of APP/PS1 Tg mice was increased by CORT treatment compared to that in vehicle-treated controls (Figures 2A,B). The plasma concentration of CORT was determined by ELISA, and the results showed that treatment with a high dose of CORT elevated the plasma levels of CORT in APP/PS1 Tg mice (Figure 2C). Moreover, the production of Aβ_1–42_ was determined by ELISA. The results showed that the production of Aβ_1–42_ in the cerebral cortex and hippocampus of APP/PS1 Tg mice was stimulated by long-term administration of CORT (Figure 2D). Therefore, these results demonstrate that GCs can enhance the production and deposition of Aβ in APP/PS1 Tg mice.

### CORT Increases the Production of Aβ by Enhancing the Expression of BACE-1 and PS1

**Figure 3:**
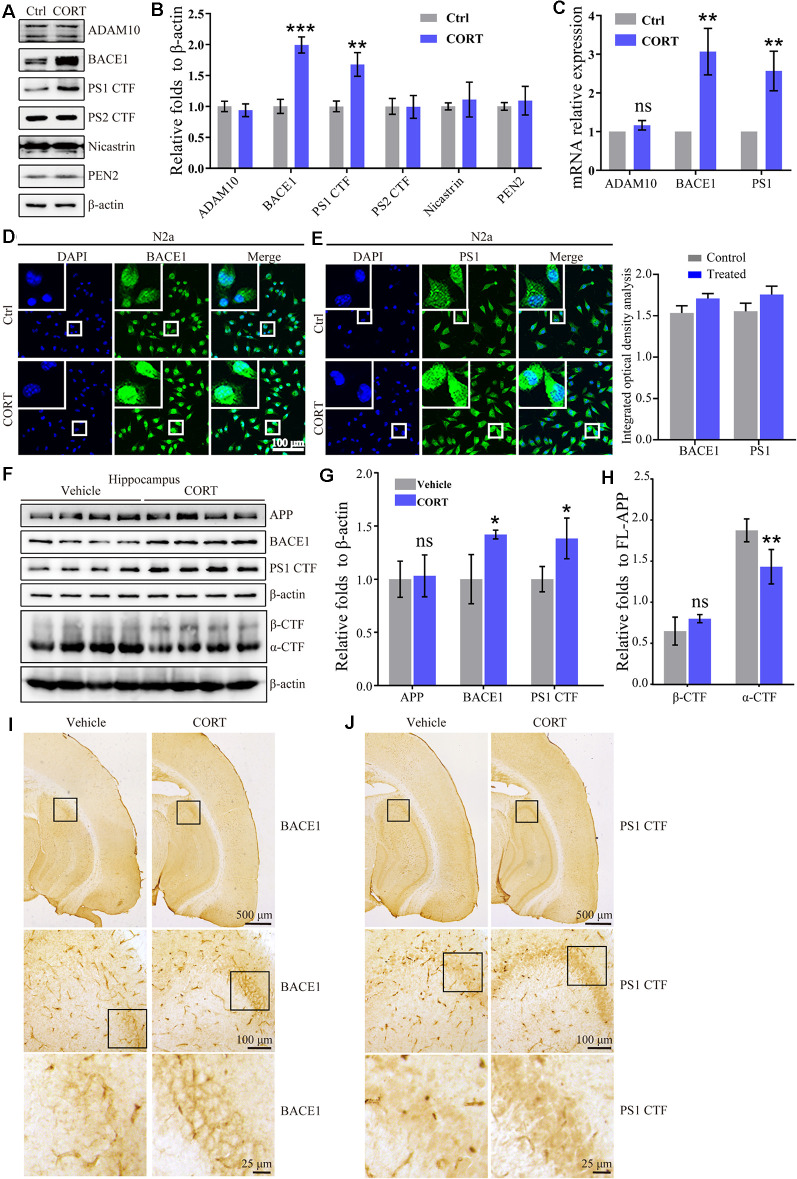
CORT treatment upregulates the expression of BACE1 and PS1 *in vitro* and *in vivo*. **(A–E)** N2a cells were treated with CORT (1 μM) for 24 h. **(A)** Western blot analysis was used to detect the expression of ADAM10, BACE1, PS1, PS2, nicastrin, and PEN2. β-actin was used as an internal control. **(B)** The optical density of the bands was analyzed by ImageJ. The data of gene expression were analyzed by the *t*-test (*n* = 6). **(C)** mRNA expression of ADMA10, BACE1, and PS1 was determined by qRT-PCR. GAPDH was used as a housekeeping gene. The gene expression data were analyzed by the *t*-test (*n* = 6). **p < 0.01; ***p < 0.001; ns: no significance compared with the vehicle-treated N2a cells. **(D,E)** The distribution of BACE1 and PS1 was detected by immunofluorescence and the immunofluorescence was semi-quantitatively analyzed by ImageJ software. **(F–I)** APP/PS1 Tg mice (5-month-old) were intraperitoneally injected with CORT (10 mg/kg/day) for 3 months. **(F–H)** The protein levels of APP, BACE1, PS1 α-CTF, and β-CTF were determined by Western blot analysis. β-Actin was used as an internal control. The optical density of the bands was analyzed by ImageJ. The gene expression data were analyzed by the *t*-test (*n* = 6). **(I,J)** The distribution of BACE1 and PS1 was determined by IHC. The data are presented as the mean ± SE of independent experiments. *p < 0.05; **p < 0.01; ***p < 0.001 compared with the vehicle-treated mice.

In the amyloid metabolic pathway, APP is sequentially cleaved by β- and γ-secretases to produce Aβ (O’Brien and Wong, 2011). To determine the mechanisms of Aβ production, the effects of CORT on the expression of α-, β- and γ-secretases was estimated. The results showed that CORT (1 μM) treatment increases the expression of the BACE1 and PS1 proteins in N2a cells (Figures 3A,B). In agreement with the results of the Western blot analysis, qRT-PCR data showed that CORT treatment upregulated the expression of BACE1 and PS1 mRNA in N2a cells (Figure 3C). The immunofluorescent imaging results confirmed the upregulation of BACE1 and PS1 in N2a cells (Figures 3D,E). Considering the *in vitro* observations, the ability of GCs to upregulate the expression of BACE1 and PS1 *in vivo* was tested. According to Western blot analysis, CORT treatment increased the expression of the BACE1, PS1, and protein level of β-CTF, while reduced the level of α-CTF without altering the protein level of APP in APP/PS1 Tg mice (Figures 3F–H). To assess the expression and distribution of BACE1 and PS1 in the brain of APP/PS1 Tg mice, sections were immunostained with antibodies against BACE1 and PS1. The results showed that the expression of BACE1 and PS1 was significantly increased in the CA3 region of the hippocampus (Figures 3I,J). Thus, CORT can promote the production of Aβ by activating BACE1 and PS1 in APP/PS1 Tg mice.

**Figure 4:**
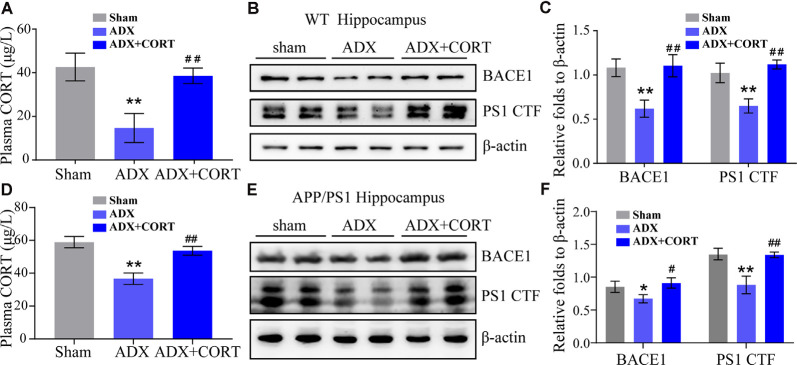
CORT is essential for the expression of BACE1 and PS1. **(A–C)** WT mice were subjected to adrenalectomy (ADX). After 3 days, mice were intraperitoneally injected with CORT (10 mg/kg/day) for 7 days. **(A)** The content of CORT was determined by ELISA. The data were analyzed by repeated-measures ANOVA (*n* = 4). **(B,C)** Protein expression of BACE1 and PS1 was determined by Western blot analysis. β-Actin was used as an internal control. The optical density of the bands was analyzed by ImageJ. The gene expression data were analyzed by one-way ANOVA (*n* = 4). **p < 0.01 compared with the sham-operated mice. ^##^*p* < 0.01 compared with the ADX-operated C57BL/6 mice. **(D–F)** APP/PS1 mice were subjected to adrenalectomy (ADX). After 3 days, mice were intraperitoneally injected with CORT (10 mg/kg/day) for 7 days. **(D)** The content of CORT was determined by ELISA. The data were analyzed by repeated-measures ANOVA (*n* = 4). **(E,F)** Protein expression of BACE1 and PS1 was determined by Western blot analysis. β-Actin was used as an internal control. The optical density of the bands was analyzed by ImageJ. The gene expression data were analyzed by one-way ANOVA (*n* = 4). The data are presented as the mean ± SE of independent experiments. *p < 0.05; **p < 0.01 compared with the sham-operated mice. ^#^*p* < 0.05, ^##^*p* < 0.01 compared with the ADX-operated APP/PS1 Tg mice.

To investigate the role of CORT in the upregulation of the expression of BACE1 and PS1, WT, and APP/PS1 Tg mice were subjected to adrenalectomy (ADX) to establish a GCs-deficient animal model. After recovery for 3 days, ADX mice were randomly divided into vehicle and CORT (10 mg/kg/day) groups and treated for 7 days. The level of CORT in plasma was determined by ELISA. The results demonstrated that ADX lowered the plasma level of CORT in WT and APP/PS1 Tg mice, which was restored by treatment of WT mice with CORT (Figures 4A,D). Protein expression of BACE1 and PS1 was always consistent with the level of CORT (Figures 4B,C,E,F), suggesting the key role of CORT in the regulation of the expression of BACE1 and PS1 in mice.

### GR Mediates the Effects of CORT on Stimulating the Expression of BACE1 and PS1

**Figure 5:**
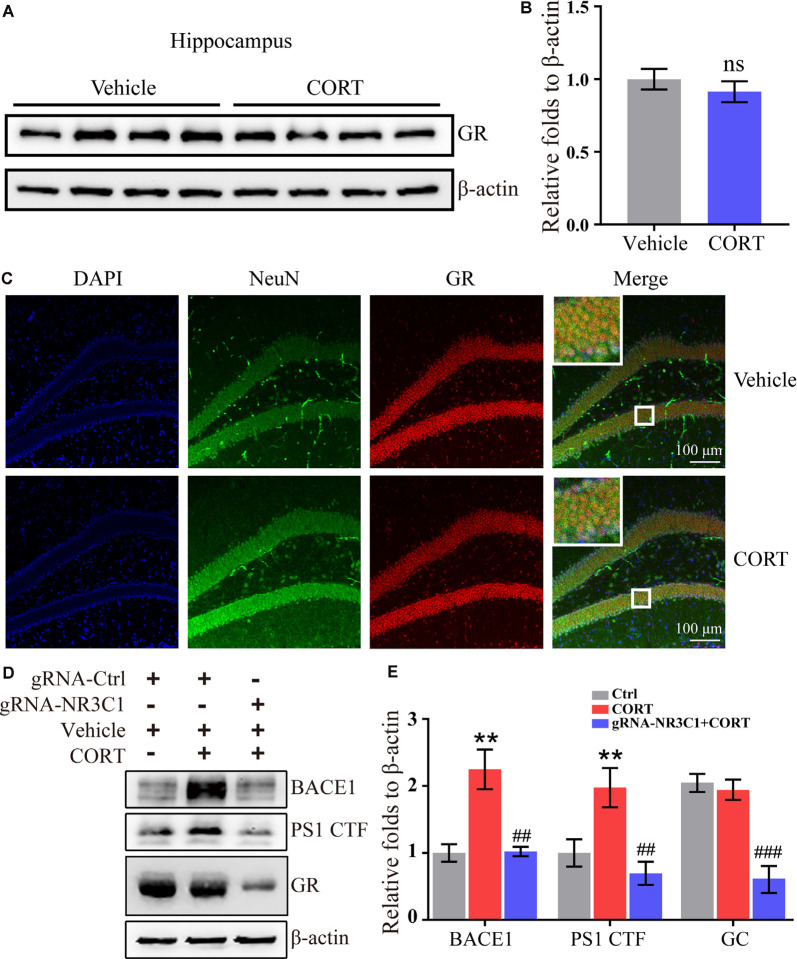
Glucocorticoid receptor (GR) mediates the effects of CORT on the upregulation of the expression of BACE1 and PS1 in neurons. **(A–C)** APP/PS1 Tg mice (5-month-old) were intraperitoneally injected with CORT (10 mg/kg/day) for 3 months. **(A,B)** The expression of the GR was determined by Western blot analysis. β-Actin was used as an internal control. The optical density of the bands was analyzed by ImageJ. The data were analyzed by the *t*-test (*n* = 6). ns: no significance compared with the vehicle-treated controls. **(C)** Brain sections of mice were double-stained with NeuN and GR antibodies and observed by confocal microscopy. **(D,E)** N2a cells were treated with CORT with or without knockdown of NR3C1 expression. Western blot analysis was used to detect the expression of BACE1, PS1, and NR3C1. β-Actin was used as an internal control. The optical density of the bands was analyzed by ImageJ. The data were analyzed by one-way ANOVA (*n* = 6). The data are presented as the mean ± SE of independent experiments. **p < 0.01 compared with the vehicle-treated controls. ^##^*p* < 0.01; ^###^*p* < 0.001 compared with the CORT-treated N2a cells.

According to the classical hypothesis of the GCs cascade, the dysfunction of the HPA axis induced by a decrease in the GR level is the initial cause of AD. Thus, the expression of the GR was determined in CORT-treated mice. The results demonstrated that treatment with CORT (10 mg/kg/day) for 3 months did not significantly lower the expression of the GR (Figures 5A,B). However, the effects of the GR on the regulation of the expression of BACE1 and PS1 cannot be neglected. To assess these effects, the distribution of the GR was determined by immunofluorescence staining. The results demonstrated that the GR colocalized with neurons (Figure 5C). Therefore, the expression of NR3C1, which encodes the GR in N2a cells (Sevilla and Pérez, 2018) was knocked down and the efficacy of knocking down was determined by Western blots. The results demonstrated that gRNA targeted NR3C1 efficiently decreased the protein levels of NR3C1. The expression of BACE1 and PS1 was attenuated by knocking down the expression of NR3C1 in CORT-treated N2a cells (Figures 5D,E). Thus, the GR mediates the effects of GCs on stimulating the expression of BACE1 and PS1 in neurons.

### CORT Upregulates the Expression of BACE1 and PS1 *via* the PKA and CREB Signaling Pathways

**Figure 6:**
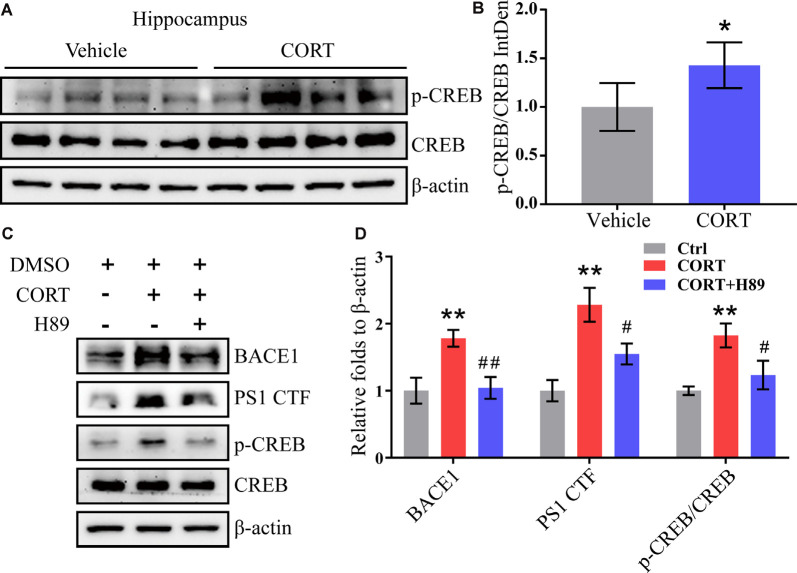
CORT upregulates the expression of BACE1 and PS1 *via* the PKA and CREB signaling pathway. **(A,B)** APP/PS1 Tg mice (5-month-old) were intraperitoneally injected with CORT (10 mg/kg/day) for 3 months. The levels of phosphorylated and total CREB were determined by Western blot analysis. β-Actin was used as an internal control. The ratio of p-CREB to CREB was calculated using ImageJ software. The data were analyzed by the *t*-test (*n* = 6). **(C,D)** N2a cells were treated with CORT (1 μM) in the absence or presence of H89 (10 μM) for 24 h. The expression levels of BACE-1, PS1, p-CREB, and CREB were measured by Western blot analysis. β-Actin was used as an internal control. The optical density was measured using ImageJ software. The gene expression data were analyzed by one-way ANOVA (*n* = 6). The data are presented as the mean ± SE of independent experiments. *p < 0.05; **p < 0.01 compared with the vehicle-treated controls. ^#^*p* < 0.05; ^##^*p* < 0.01 compared with CORT-treated N2a cells.

CREB is an important molecule involved in the regulation of memory in AD and was thus included in the current study to determine the mechanisms of its effects (Bartolotti and Lazarov, 2019). APP/PS1 mice were intraperitoneally injected with CORT at a dose of 10 mg/kg/day for 3 months. The Western blot analysis results indicated that phosphorylation of CREB was elevated in CORT-treated APP/PS1 Tg mice (Figures 6A,B). To validate these observations, N2a cells were treated with CORT (1 μM) in the absence or presence of a PKA/CREB inhibitor, H89 (10 μM). After 24 h, phosphorylation of CREB was blocked by H89 in CORT-stimulated N2a cells (Figures 6C,D). Deactivation of CREB resulted in attenuation of the effects of CORT on the stimulation of the expression of BACE1 and PS1 (Figures 6C,D). These results suggest the PKA and CREB signaling pathways are involved in mediating the effects of CORT on the upregulation of the expression of BACE1 and PS1.

### CORT Promotes Apoptosis of Neurons and Inhibits Neuronal Differentiation in the Brain of APP/PS1 Tg Mice

**Figure 7:**
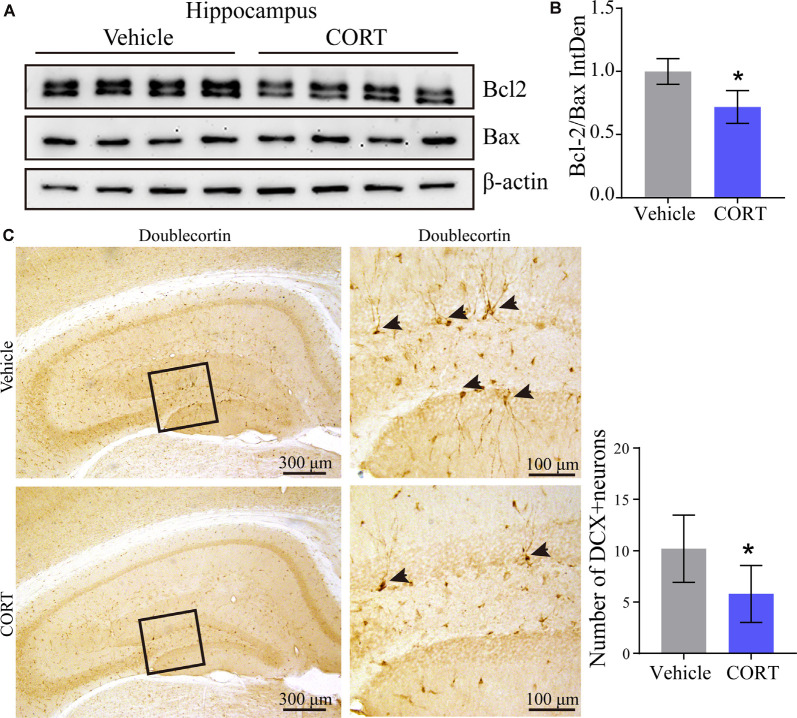
CORT promotes apoptosis of neurons and inhibits neuronal differentiation in the brain of APP/PS1 Tg mice. APP/PS1 Tg mice (5-month-old) were intraperitoneally injected with CORT (10 mg/kg/day) for 3 months. **(A,B)** Western blot analysis was used to detect the expression of Bcl-2 and Bax in the hippocampus of mice in each group. β-Actin was used as an internal control. The bands were analyzed using ImageJ software. The data were analyzed by the *t*-test (*n* = 6). **(C)** Neuronal differentiation was determined by IHC staining of doublecortin. The positive neurons were semi-quantitatively analyzed by ImageJ software. The data are presented as the mean ± SE of independent experiments. *p < 0.05 compared with the vehicle-treated controls.

Our results identified the mechanisms of action of CORT in the regulation of the production and deposition of Aβ; thus, the roles of CORT in neurons were further investigated. A series of studies have suggested the neurotoxic effects of Aβ on apoptosis of neurons (Reddy and Beal, 2008; Calvo-Rodriguez et al., 2019). To determine the effect of CORT on the brain of APP/PS1 Tg mice and to assess whether CORT can promote apoptosis of neurons in the brain, Western blot analysis was used to detect the expression of Bcl-2 and Bax in the hippocampus in all groups of mice. The results showed that the ratio of Bcl-2 to Bax was significantly downregulated in the brain of CORT-treated APP/PS1 Tg mice compared with that in the control groups (Figures 7A,B). This observation suggests that CORT promotes apoptosis of neurons in the brain of APP/PS1 transgenic mice. Since CORT induces apoptosis of neurons, the roles of CORT in neuronal differentiation were determined by staining doublecortin. The results demonstrated that axons and dendrites generated from neuronal stem cells are suppressed by treatment with CORT (Figure 7C). These results indicate that CORT can aggravate AD *via* multiple mechanisms, such as induction of the production and deposition of Aβ, triggering apoptosis of neurons, and inhibiting neuronal differentiation.

## Discussion

As the involvement of GCs in regulating the learning and cognitive functions (Starkman et al., 2001, 2003), we investigated the effects of GCs on AD. As a consequence, we found that the content of CORT in plasma was significantly increased in the 9-month-old APP/PS1 Tg mice. By subcutaneously injected CORT, the learning ability of APP/PS1 Tg mice was impaired with enhancing the production and deposition of A in a BACE1- and PS1-dependent mechanism. To the mechanism, CORT activates BACE1 and PS1 *via* the glucocorticoid receptor (GR)-PKA-CREB signaling pathway. These observations were further confirmed by ADX treatment, which downregulates the expression of BACE1 and PS1 by reducing the plasma levels of CORT.

Prior studies have shown that stress can accelerate brain aging, which results in the induction of dementia (Brinks et al., 2007). Additionally, stress has an impact on the major depressive disorder (MDD; Roy et al., 2017). Accumulating evidence shows that patients with MDD have cognitive disorders with symptoms similar to AD patients (Boedeker et al., 2020). These observations define a potential link between stress, MDD, and AD (Galts et al., 2019). Specifically, stress, MDD, and AD share the same characteristic of overproduction of GCs. Long-term exposure to a normal concentration of GCs accelerates brain aging, which results in a loss of the ability to regulate the levels of GCs, thus leading to further acceleration of brain aging and, eventually, causing brain impairment and onset of dementia. However, it is unclear whether GCs leads to the onset and development of AD or plays a role in AD-associated mechanisms. Certain controversies regarding the specific molecular mechanisms require additional clarification despite numerous studies on these issues. Therefore, the roles of CORT in the regulation of the development and progression of AD were investigated in the present study. The results indicate that CORT has multiple pathological functions in the progression of AD, including regulation of the production and aggregation of Aβ, apoptosis of neurons and suppression of neuronal differentiation.

Clinical data show that the average daily fluctuations of cortisol levels in elderly people are higher than those in younger people (Raskind et al., 1994). In the last century, elevated plasma levels of cortisol have been reported to induce a reduction in the volume of the hippocampus and impairment of memory in elderly people. Certain patients with high cortisol levels progressively develop AD (Lupien et al., 2005). Similarly, GCs are also responsible for a decrease in the volume of the hippocampus and memory impairment in patients with Cushing’s syndrome (CS; Forget et al., 2000). In agreement with these observations, the level of cortisol is significantly higher in AD patients than in the control subjects (Raskind et al., 1982). Also, epidemiological studies have revealed that high levels of cortisol can upregulate the expression of AD-associated genes and can be used to diagnose AD *via* long-term monitoring of the level of cortisol (Ennis et al., 2017). The plasma concentration of CORT in 3×Tg AD mice was significantly higher than that in WT mice (Green et al., 2006). Thus, the prior studies have been expanded and it has been demonstrated that the plasma concentration of CORT is elevated in 9-month-old APP/PS1 Tg mice compared to that in WT mice.

However, overloading of GCs is not always bad for brain aging. For example, acute and transient secretion of GCs is beneficial to some types of learning and memory, such as emotional ability (McGaugh and Roozendaal, 2002) and spatial memory (Wingenfeld and Wolf, 2014). In contrast, a series of studies have demonstrated that GCs can increase the levels of Ca^2+^-mediated electrophysiological biomarkers of hippocampal aging in the range from a few minutes to several hours (Kerr et al., 1989, 1992; Joëls and de Kloet, 1993). Therefore, the effects of short-term exposure to GCs on learning and memory remain a matter of debate. However, high levels of GCs or long-term exposure to a normal concentration of GCs will continuously activate the GR, which results in damage to memory (Zhang et al., 2020). In a preliminary study in the salmon and mammalian (including human) brain, an increase in GCs resulted in the degeneration of peripheral tissues, similar to the changes associated with aging (Sotiropoulos et al., 2008). In an ADX group of rats, several biomarkers of brain aging were significantly reduced, resulting in improved learning and memory performance in the Morris maze test compared with that in the sham group (Landfield et al., 1981). GCs have been shown to damage the hippocampus by increasing its vulnerability (Sapolsky et al., 1986). In detail, GCs impairs the energy metabolism of hippocampal neurons by inhibiting the uptake of glucose, which results in an increase in the susceptibility of cultured neurons to all types of destructive metabolic damage (Sapolsky et al., 1988). Additionally, CORT defines the relationship between hippocampal damage and cognitive impairment in elderly rats (Issa et al., 1990; Meaney et al., 1995), which is supported by other reports that demonstrated a negative correlation between GCs exposure and cognitive decline. On the other hand, excessive loading with GCs will cause atrophy of hippocampal dendrites and delay neurogenesis (McEwen, 1996; Mirescu and Gould, 2006). Based on these studies, CORT treatment-induced cognitive decline in APP/PS1 Tg mice.

Secretion of GCs is regulated by the HPA axis. The GR is the key molecule of the HPA axis that regulates the level of GCs *in vivo*. Under pathological conditions, the concentrations of GCs increases *in vivo*. High levels of GCs act on the GR in the hippocampus, hypothalamus, and pituitary, which in turn, induce a decrease in GCs to the basal level *via* a negative feedback mechanism. Release of GCs leads to downregulation of the expression of the GR and its downstream signaling pathway and atrophy and loss of GR-containing neurons in the hippocampus, which suppresses the regulatory effects of the GR on the HPA axis (Sotiropoulos et al., 2008). The mRNA and protein levels of the GR are decreased with age in the hippocampus, damaging the negative feedback regulation of GCs and leading to continuous exposure of the brain to high concentrations of GCs, which results in loss of neuronal function (Landfield, 1978; Landfield et al., 1978; Jacobson and Sapolsky, 1991). Without chronic GCs stress, overexpression of the GR in the mouse forebrain has been reported to be responsible for the acceleration of the expression of brain aging-like phenotype (Wei et al., 2007). Also, subchronic inhibition of the GR in AD model mice can treat early defects of situational memory and synaptic plasticity (Lanté et al., 2015). The GR is a nuclear receptor that acts as a transcription factor to mediate the effects of CORT on the upregulated expression of BACE1 and PS1. GCs bind to the GR in the cytoplasm, which then enters the nucleus to regulate the transcription of APP and BACE1 *via* binding to the CRE sequence of the APP and BACE1 promoters (Green et al., 2006). The present study expands on these previous findings by demonstrating that knockdown of expression of NR3C1 blocks the effects of CORT on stimulating the expression of BACE1 and PS2, suggesting that CORT upregulates the expression of BACE1 and PS1 *via* the GR in neurons.

CREB is a transcription factor that plays an important role in regulating the transition from short-term memory to long-term memory (Tully et al., 2003; Saura and Valero, 2011). Additionally, the CREB signal is involved in the regulation of processes associated with many neurodegenerative diseases, such as AD. For example, CREB has been reported to regulate the expression of BACE1 and PS1 as a transcription factor (Lahiri et al., 2006; Choi et al., 2017). The results of the present study demonstrated that CORT can stimulate the phosphorylation of CREB at Ser 133, which results in upregulation of the expression of BACE1 and PS1 in neurons. The addition of H89 completely blocked the effects of CORT on stimulating the expression of BACE1 and PS1 *via* dephosphorylation of CREB, suggesting the key role of CREB in mediating the effects of CORT on regulating the expression of BACE1 and PS1 in neurons.

According to Aβ theory, APP is abnormally cleaved to produce Aβ, which can induce apoptosis of neurons *via* multiple pathways (Reddy and Beal, 2008; Calvo-Rodriguez et al., 2019). Aβ can concurrently upregulate the expression of Bax and downregulate the expression of Bcl-2, thus disrupting the balance between Bax and Bcl-2, leading to apoptosis of neurons during the course of AD development and progression (Hu et al., 2016). The results of the present study indicate that CORT can induce the production of Aβ and decrease the ratio of Bcl-2 to Bax, suggesting that CORT promotes apoptosis of neurons. Moreover, CORT inhibits neuronal differentiation in APP/PS1 Tg mice. In agreement with these results, chronic exposure to high concentrations of CORT has been shown to reduce neuronal differentiation in the dentate gyrus of rats (Brummelte and Galea, 2010). Inhibition of the secretion of CORT from midlife to the rest of the animal life increases neuronal differentiation in old animals, which prevents the onset of age-related memory disorders (Montaron et al., 2006). Thus, the effects of CORT trigger a loss of neurons during AD development and progression.

For neuroinflammation, more and more data proved the crosstalk between neuroinflammation and Aβ, which can accelerate the progression of AD (Cai et al., 2014). Since studies have shown that GCs induce the expression of nod-like receptor family in cultured macrophages and primary macrophages, leading to the secretion of pro-inflammatory cytokines IL-1, TNF-α, and IL-6 (Busillo et al., 2011), we continue to detect if CORT can regulate the neuroinflammation of APP/PS1 Tg mice. As the main cell types that mediate neuroinflammation in the brain are astrocytes and microglia (Sawikr et al., 2017), we immunostained the glial cells with GFAP and Iba1. The results demonstrated that CORT activates the glial cells of APP/PS1 Tg mice, leading to the secretion of TNF-α (data not shown). In agreement with our results, CORT has been reported to activate microglial cells (Zalewska et al., 2017) and astrocytes (Bridges et al., 2008). Moreover, intravenous infusion of CORT at non-stressed (35 ng/ml) and stressed levels (350 ng/ml) increased the release of TNF-α and/or IL-6 in the liver (Liao et al., 1995).

Apart from the above effects, high GCs can inhibit mTOR-dependent autophagy, leading to the aggregation and deposition of tau, which results in neuronal death of Tau^P301L^ mice (Silva et al., 2019). Also, GCs in the brain are the key regulators of dendritic spines. For example, Pedrazzoli et al. (2019) found that dexamethasone, an agonist of GRs, can significantly reduce the density of dendritic spines in the hippocampal CA1 area of 6- and 10-month-old 3×Tg-AD mice. Moreover, recent work suggests that GCs may regulate synaptic plasticity by interacting with glutamate-energy mechanisms and ultimately affect learning and memory processes (Sandi, 2011).

## Conclusions

Elevated levels of CORT during the course of AD development and progression can induce the production and deposition of Aβ in APs by activating BACE1 and PS1 in APP/PS1 Tg mice. Additionally, the GR mediates the effects of high dose CORT exposure by stimulating the expression of BACE1 and PS1 *via* the PKA and CREB signaling cascades. These mechanisms of CORT promote apoptosis of neurons and inhibit neuronal differentiation, resulting in cognitive decline in APP/PS1 Tg mice.

## Data Availability Statement

The raw data supporting the conclusions of this article will be made available by the authors, without undue reservation.

## Ethics Statement

The animal study was reviewed and approved by medical laboratory animals (Ministry of Health, Peoples Republic of China, 1998) and the laboratory animal ethical standards of Northeastern University of China were adhered to.

## Author Contributions

S-QZ and L-LC conceived and performed all of the experiments, participated in the design of the study, and wrote the manuscript. Y-YL carried out select experiments. PW interpreted the data and wrote the manuscript. All authors contributed to the article and approved the submitted version.

## Conflict of Interest

The authors declare that the research was conducted in the absence of any commercial or financial relationships that could be construed as a potential conflict of interest.
