# Supplementary figures and images for: The Molecular Mechanism of Chronic High-Dose Corticosterone-Induced Aggravation of Cognitive Impairment in APP/PS1 Transgenic Mice
Source: Front Mol Neurosci. 2021 Jan 15;13:613421. doi: 10.3389/fnmol.2020.613421 (PMC7844096; doi:10.3389/fnmol.2020.613421)

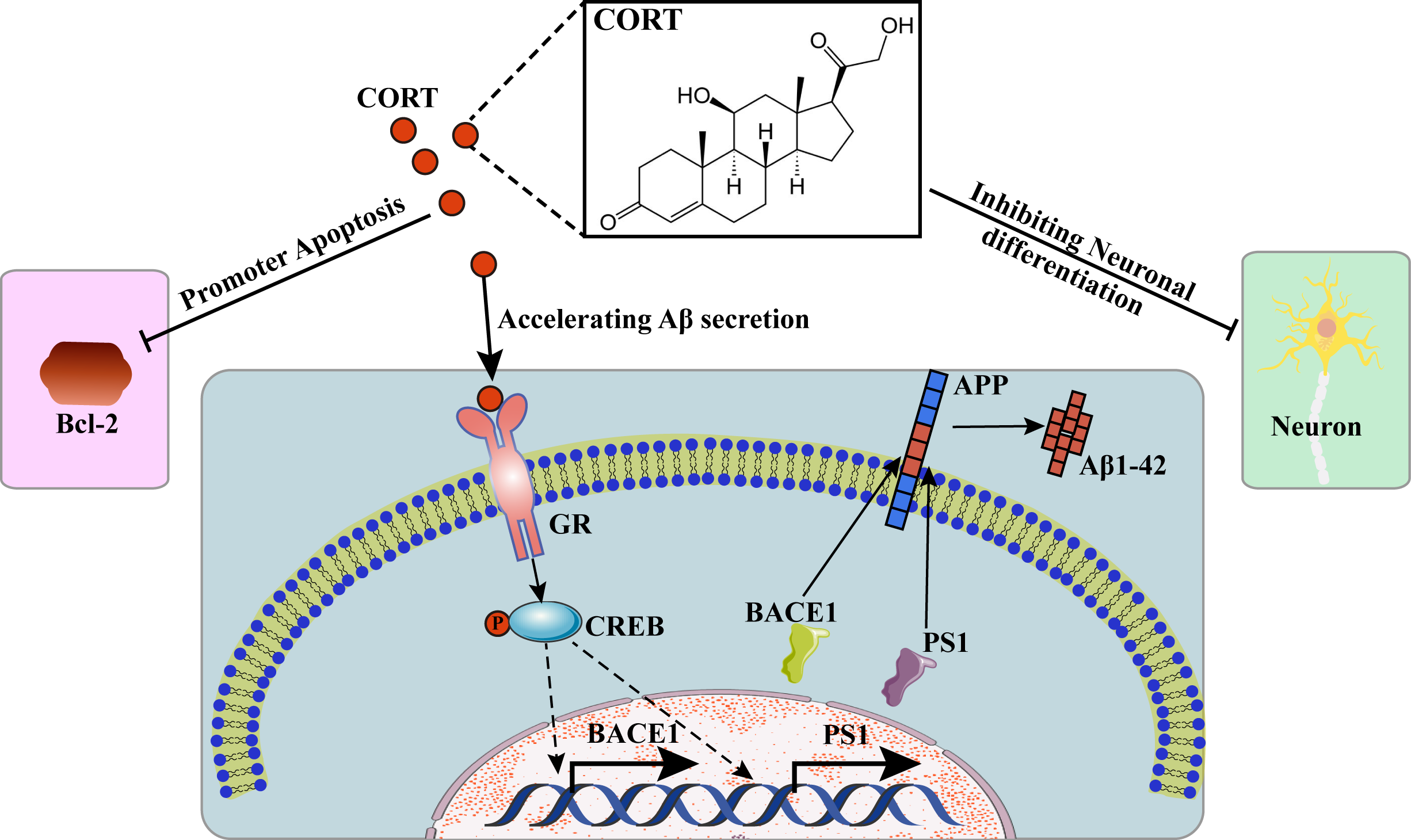

Supplement: Supplementary file 1 [file Image_1.TIF]
